# A virtual reality interface for the immersive manipulation of live microscopic systems

**DOI:** 10.1038/s41598-021-87004-5

**Published:** 2021-04-07

**Authors:** Stefano Ferretti, Silvio Bianchi, Giacomo Frangipane, Roberto Di Leonardo

**Affiliations:** 1 Soft and Living Matter Laboratory, NANOTEC-CNR, Institute of Nanotechnology, 00185 Rome, Italy; 2 Physics Department, Sapienza University of Rome, 00185 Rome, Italy

**Keywords:** Microscopy, Optical manipulation and tweezers

## Abstract

For more than three centuries we have been watching and studying microscopic phenomena behind a microscope. We discovered that cells live in a physical environment whose predominant factors are no longer those of our scale and for which we lack a direct experience and consequently a deep intuition. Here we demonstrate a new instrument which, by integrating holographic and virtual reality technologies, allows the user to be completely immersed in a dynamic virtual world which is a simultaneous replica of a real system under the microscope. We use holographic microscopy for fast 3D imaging and real-time rendering on a virtual reality headset. At the same time, hand tracking data is used to dynamically generate holographic optical traps that can be used as virtual projections of the user hands to interactively grab and manipulate ensembles of microparticles or living motile cells.

## Introduction

During the second half of the 17th century, scientists like Hooke and van Leeuwenhoek revealed to the world the beauty and complexity of a microscopic universe that was made visible for the first time by microscope lenses. Centuries of developments in optical design have perfected optical hardware to produce detailed and aberration free images. In more recent years, lasers, digital cameras, spatial light modulators and consumer high performance computing are transforming the optical microscope into an active investigation tool with ever expanding possibilities for interactions with microscopic systems. Using optical tweezers^1–3^ we can trap individual cells^4^, measure their mechanical stiffness^5^ or arrange them in precisely controlled 3D micro-environments^6^. Although a few advanced interfaces have been proposed to replace the computer mouse with a multi-touch interface^7^ or force-feedback devices^8^, the visual feedback has always been limited to 2D projections viewed through the window of a computer display. Computational approaches to microscopy allow high frame volumetric imaging by numerical reconstructions based on 2D holographic patterns that encode the full 3D structure of the scene^9–14^. On a parallel track, virtual reality (VR), augmented reality and mixed reality are transforming the way we explore and acquire information from the macroscopic world around us. Applications in science are still very limited but potentially transformative in many fields. Neuroscientists are increasingly using virtual reality to explore the inner workings of animal and human brains^15^ by simulating real world inputs. In the context of molecular dynamics simulations, virtual reality interfaces are found to facilitate sophisticated molecular modeling tasks^16^. It has also been shown that virtual reality can play an important role in scientific education and training, allowing safe and economical experiments to be performed in virtual laboratories^17^. In all of these applications, however, virtual reality is doubly artificial being a software rendering of software objects. More than simulating presence in artificial environments, using virtual reality we can be virtually present in real but physically unaccessible worlds to explore and manipulate them from “within”. This idea of teleoperation in the nano and micro world was first put forward using the Scanning Tunneling Microscope (STM)^18^. By connecting a haptic device to the scanning tip of a STM and using a virtual reality headset to render topographic reconstructions, the user could either “fly” over the surface or “feel” the surface under her hand or deposit gold mounds over it^19^. Following this pioneering work, the field of telenanorobotics has shifted towards the use of the Atomic Force Microscope (AFM)^20^ which offers the advantages of being more suitable for a wider range of samples, including biological cells and macromolecules, and also to provide direct force measurements for tactile feedback. Using a haptic device and 3D topographic reconstructions for visual feedback, it is possible to manipulate strands of DNA, nanotubes and other macromolecules that lie on a surface^21^. However, the use of a scanning tip involves a few drawbacks: (1) imaging and manipulation need the same tip and cannot be simultaneous, (2) 3D reconstructions are slow and limited to topographic surfaces preventing applications to objects that float and move in a 3D space, (3) manipulation is limited to “pushing with the tip” and although more complex operations could be performed using two tips for grabbing, this is actually slow, cumbersome and invasive^22^. Here we introduce a novel approach to tele-micromanipulation that is fully based on optical tools for simultaneous 3D visualization and real-time manipulation of micro-systems through an immersive Virtual Reality interface. Through this instrument we can be virtually “shrunk ” by a million times and sent inside a sealed microscope slide where we can grab swimming cells on the fly and use our hands to interactively arrange colloidal particles in precise 3D geometric configurations. A 3-axis implementation of holographic microscopy^23,24^ allows the real-time reconstruction of a 3D scene that is the simultaneous replica of a real microscopic sample sitting under a microscope in a separate laboratory room. Using hand tracking devices we project our hands in this virtual micro-reality and use them to grab and move microscopic objects by means of optical traps that are reconfigured in real time based on hand tracking data. We report a series of exploratory experiments with microscopic systems composed of both synthetic microspheres and live swimming bacteria. Further developments in computational microscopy will allow direct interaction with more complex objects like larger eukaryotic cells or complex micromachines^25^ which could be assembled and operated in an immersive environment.

**Figure 1:**
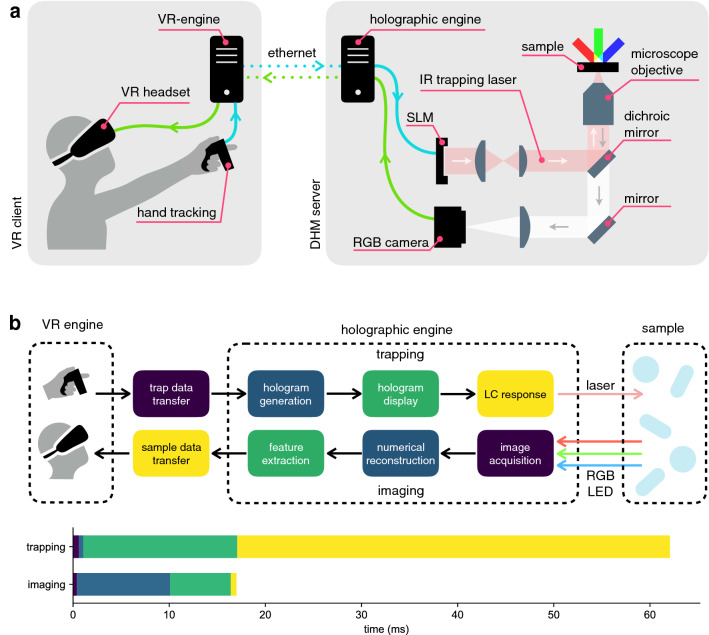
(**a**) Schematic view of the optical and computer hardware setup. The sample is illuminated by three tilted RGB LEDs and a 100$\times$ magnified image is captured by a color camera. The “holographic engine”, running on a control PC in the lab, performs 3D numerical reconstructions and identifies the objects in the field of view. Position and shape parameters of the objects are sent to a remote PC running a “VR-engine” that renders the objects on a VR headset. Hand tracking input data is transferred back to the “holographic engine” for the computer generation of digital holograms to be displayed on the SLM. The SLM shapes the wavefront of an IR laser beam that is then focused by the same imaging objective to generate the desired 3D traps arrangement. (**b**) Data and optical flow diagram and related timing chart. Chart colors correspond to the boxes above it. This figure was made with Keynote^26^.

## Results

### A virtual interface to micro-reality

The use of a virtual reality environment as a live interface for a microscopic system requires the ability to perform interactive 3D manipulations while simultaneously observing a 3D real-time reconstruction of the scene being manipulated. On the one hand, holographic tweezers^3^ offer a powerful and mature technology for multi-particle, 3D manipulation of micron sized objects. On the other hand, latest implementations of digital holographic microscopy (DHM) allow a high frame rate volumetric reconstruction with a good resolution in the three directions of space. These trapping and microscopy techniques are called “holographic” since in both cases a 3D structure is encoded in a 2D light field that is the principle of holography. In the case of holographic trapping, a collection of laser spots is created in 3D space by modulating the light field on the Spatial Light Modulator (SLM) plane, while, in holographic microscopy, a volumetric light distribution is obtained from a 2D interference pattern recorded by the camera. We integrate these two technologies through a virtual reality interface as schematically illustrated in Fig. 1. Based on hand tracking data, digital holograms are dynamically generated on a computer and displayed on a SLM. The SLM shapes the wavefront of a collimated laser beam in such a way that, after propagation through a microscope objective, an array of focal spots is generated in a 3D volume around the focal plane. Each of these spots provides an independent optical trap that can be used to grab and move a micron sized object such as a colloidal particle or a living cell.

**Figure 2:**
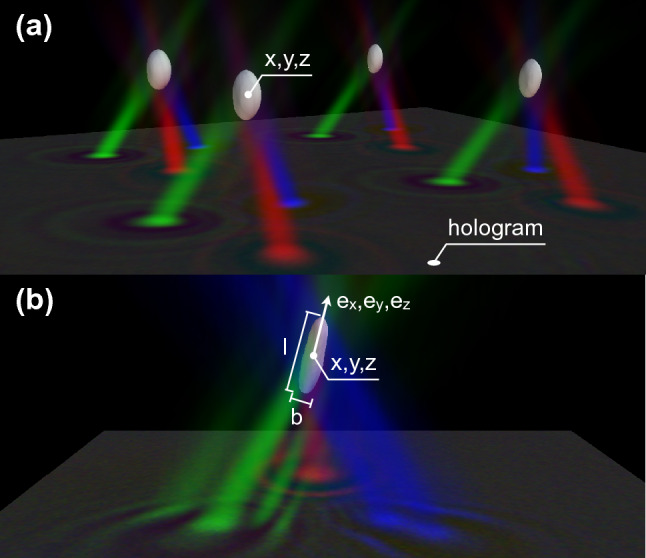
Holographic reconstructions of colloidal spheres and *E. coli* bacteria. From each color channel in a single 2D color hologram we obtain numerically the three independent scattered fields whose intensities are represented by red, green and blue density maps. An isosurface of the overlapped intensity is represented in white and closely matches the shape of simple microscopic objects such as colloidal spheres (**a**) or *E. coli* bacteria (**b**). Morphological analysis of these overlap regions gives information on position (*x*, *y*, *z*), size and shape (*l*, *b*) and orientation ($e_x,e_y,e_z$) of the objects. This figure was made using Mayavi^27^.

**Figure 3:**
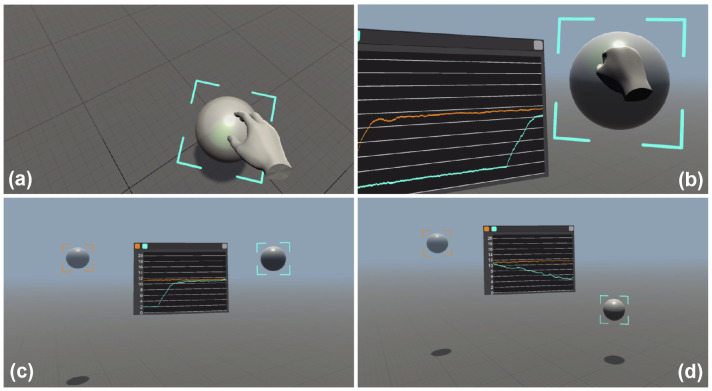
A silica (right) and a polystyrene (left) microspheres, both having a radius of 1 $\upmu$m, are grabbed with a virtual hand (**a**), lifted to a height of $z=10\,\upmu$m from the coverslip (**b**) and finally released to observe their sedimentation (**c**). The heights of the targeted particles can be monitored in real time on a chart that clearly shows that the right particle is heavier (**d**). See also Supplementary Video 1. This figure was made using Unity^28^.

Exploiting the computational power of modern Graphics Processing Units (GPU), optimized digital holograms can be generated in a few milliseconds and follow user input with a negligible time lag^29^. Although it is possible to manipulate multiple objects in 3D and dynamically following user input^7,29,30^, the visual feedback we get is typically limited to a 2D slice of the sample scene, obtained by bright field or epifluorescence microscopy^7,31^. Three dimensional reconstructions of optically trapped particles can be produced by confocal microscopy^32^ but they are usually much slower than video rate due to the need for mechanical focus scanning. An alternative 3D and label-free imaging tool is diffraction tomography that allows for a 3D reconstruction of the sample refractive index from stacks of 2D frames acquired while changing the illumination angle^9–11,13,14^ or while scanning the focal plane position along the optical axis^12^. These frames stacks could be acquired with a real-time framerate, however, diffraction tomography is computationally very intense and consequently too time consuming to be used for real-time visualization of the sample. In this respect, direct numerical back propagation of scattered field is preferable. This method, which is usually referred to as in-line holographic microscopy^33–35^, combines a high speed acquisition rate with relatively light computational load at the cost of having volumetric images with a poor axial resolution. We recently introduced an implementation of in-line holographic microscopy that significantly improves the resolution along the optical axis (lateral and axial resolution, respectively: 0.4 $\upmu$m and 0.8 $\upmu$m)^23,24^. Three collimated LEDs, each having a different color (RGB), impinge on the sample from three different directions. Each channel of a digital color camera records an independent hologram produced by the interference between incident light and the light scattered by the object. These three digital images are transferred to a GPU device where three corresponding volumetric reconstructions are calculated using the Rayleigh-Sommerfeld back propagation^36^. Although each of those reconstructions suffers from poor axial resolution, their overlap returns a volumetric image whose isosurface contours represent quite closely the surface of simple microscopic objects such as colloidal spheres^23^ or *E. coli* bacteria^24^ as shown in Fig. 2. We integrated these two technologies using a game engine that supports user interaction through VR devices as schematized in Fig. 1). Optical traps are implemented as game objects that can be interactively and independently created, destroyed, selected and moved around using hand gestures (*i.e.* “grab”) or more sophisticated remote control tools. When the engine detects an event related to the creation, destruction or displacement of traps, the data describing the updated traps configuration is sent through an ethernet connection to the “holographic engine”. The “holographic engine” runs on a computer controlling the optical hardware in a separate lab. When an update request is received from the “VR engine” the “holographic engine” computes an optimized digital hologram on the GPU and directly displays it on a liquid crystal spatial light modulator. A collimated infrared laser beam ($\lambda$=1064 nm) reflects off the SLM and acquires a phase modulation such that, after propagation through a microscope objective (Nikon 100x, Plan Apo $\lambda$, NA=1.45) produces diffraction limited spots having a spatial arrangement that is the same of their virtual counterparts. Each of these spots serves as an optical trap that can be used to grab and manipulate small dielectric objects, such as the colloidal beads in Fig. 3, in a 3D region covering the entire field of view. Typically trap rearrangements will result in the rapid motion of nearby objects which are captured in holographic images at 40 fps and processed in real time by the “holographic engine”. The obtained volumetric reconstructions are segmented to extract the relevant geometric features of all identified objects. These geometric data are sent back to the “VR engine” to update the geometric parameters of game objects providing a virtual representation of the real objects that are being interactively manipulated under the microscope. This closed complex flow of data and light starting from the “VR engine” is summarized in Fig. 1b. For trapping alone, the maximum achievable refresh rate is limited to 20 Hz the main bottleneck being the slow response dynamics 45 ms of the SLM liquid crystals. Even if we neglect this slow response the SLM is refreshed at a 60 Hz rate corresponding to a minimum lag time for hologram display of 17 ms. Optimized phase masks are generated using a GSW algorithm^29,30^ with a computation time that scales linearly with the number of traps as well as the number of optimization steps. We set the number of GSW iterations to 3 giving an expected efficiency (ratio of trapping spots power over the total power) of about 80%. The resulting total computation time is 0.48 ms per trap so that most of the computational time (blue bars in Fig. 1b) is used for image analysis. On the imaging side, the main framerate limiting factor is the computational time of the tasks performed by the “holographic engine”. Starting from a 512$\times$512 RGB frame, whose acquisition time is negligible, we obtain a volumetric image of 512$\times$512$\times$61 voxels (corresponding to a field of view of $56 \times 56\times 20\,\upmu$m$^3$) in 9.7 ms. The segmentation of the volumetric image takes about 5.8 ms while the extraction of position and orientation of each of the identified objects takes about 0.5 ms. For instance, if two objects are present in the field of view as in Fig. 3, the total time required to obtain a full geometric description of the scene is 16.5 ms. System latency, i.e. the time interval between a trap rearrangement input from hand tracking devices and the actual observation of the resulting beads motions, can be estimated from data in Fig. 1 by summing the total trapping time (62 ms) to the total imaging time (16.5 ms) which gives an overall latency of about 80 ms. This latency is low enough to guarantee a smooth and interactive manipulation experience. In order to match coordinates between trapping and imaging space, we trap a glass microsphere (1 $\upmu$m radius) and scan its position ${\mathbf{r}}^\prime$ in trapping space on a three dimensional grid. During scanning, the trapping laser power is maximum to minimize displacements from the trap’s equilibrium position caused by Brownian fluctuations and gravity. We extract the corresponding bead’s coordinates ${\mathbf{r}}=(x,y,z)$ in imaging space from the 3D reconstructions. Finally we compute the transform ${\mathbf{r}}^{\prime }=T {\mathbf{r}}+{\mathbf{r}}_{0}+\alpha z^2{\hat{\mathbf{z}}}$ by fitting the matrix *T*, a linear transform operator, the vector ${\mathbf{r}}_{0}$ which represents the displacement between the two origins and the empirical factor $\alpha$ which takes into account small displacements in the position of the trap due to spherical aberrations.

### Application example 1: falling bodies

As a first application we choose a simple experiment that demonstrates the potential impact of our instrument for physics education. If we grab two balls, one made of plastic and the other one made of glass and release them from the same height, they will accelerate with constant speed along the vertical direction and hit the ground at approximately the same time. Now we can do the same experiment but on a microscopic scale. As shown in Supplementary Video 1 (representative frames in Fig. 3) we can virtually walk over a microscope coverslip, look around and find two spheres, this time having a radius of 1 $\upmu$m, floating above it. Using our own virtual hands we can grab (Fig. 3a), lift (Fig. 3b) and release them (Fig. 3c) and then watch them falling in front of our eyes (Fig. 3d). We can directly experience that a falling body experiment on the microscale looks very different that on the macroscale. The spheres do not move on a vertical line but have a strong Brownian random motion. Although they slowly sediment, it often happens to see them moving up against gravity which constitutes an apparent violation of the second law of thermodynamics^37^. Our interface also implements a series of tools to track objects and watch the time evolution of their coordinates on a display. The video shows that two balls have very different sedimentation speeds with the glass ball drifting down with a higher mean speed. All this can be explained to students with words, pictures and movies but doing the experiment in first person and watching all this happening in front of our eyes offers a unique and powerful experience of the physical laws that govern the micro-world populated by cells and colloidal particles.

**Figure 4:**
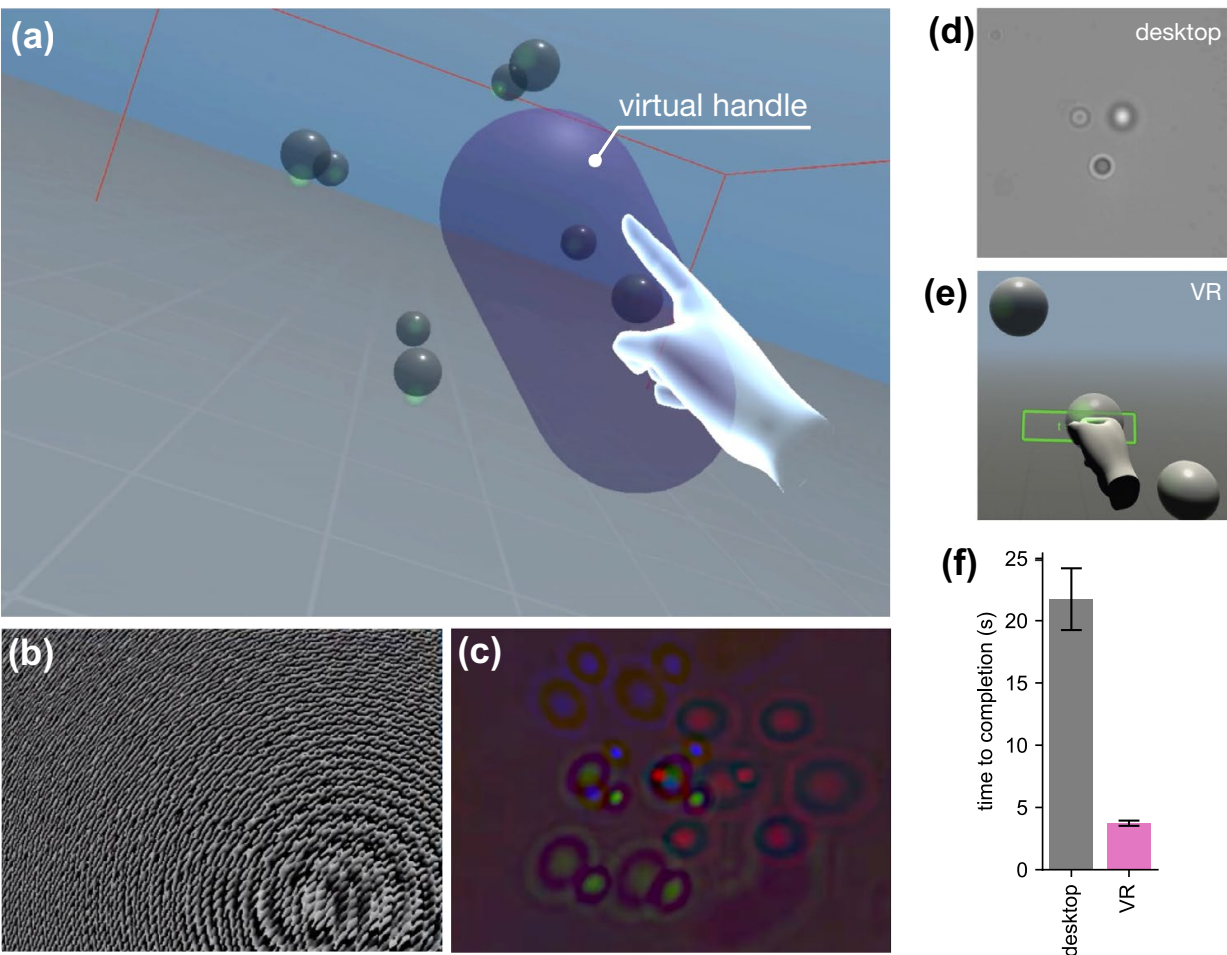
(**a**) Eight silica microspheres of 2 $\upmu$m diameter are interactively arranged on the vertices of a cube and then rigidly translated and rotated through a virtual global handle in purple color. (**b**) The optimized phase mask that is displayed on the SLM to generate the trap arrangement in (**a**). (**c**) RGB hologram recorded by the camera and processed in real-time to obtain the scene in (**a**) as described in Fig. 2. See also Supplementary Video 2. (**d**) User microassembly task using the standard desktop interface. (**e**) User microassembly task through our VR interface. (**f**) Barplots represent the sample mean of time to completion using the two interfaces (error bars are standard errors). This figure was made using Unity^28^ and Matplotlib^38^.

### Application example 2: handcrafting microstructures

The primary advantage of using holographic optical tweezers is the ability to dynamically arrange multiple traps in 3D. With holographic optical tweezers we can precisely arrange multiple colloidal particles or living cells in controlled spatial configurations to iteratively study their stochastic behavior following reproducible initial conditions^24,39^ or their biological interactions during growth^6^. Using multiple traps it is also possible to grab and rotate microfabricated objects with complex shapes which can be used as tools for advanced microscopy applications^31,40^ or for the study of Brownian motion and hydrodynamics of non spherical objects^41,42^. In this regard, a virtual reality interface could simplify and accelerate the assembly of a multi-component microsystem^43^ and allows its direct operation with hand gestures and immersive feedback in real time. As a demonstration of this we show in Fig. 4 the live manipulation of a cubic structure made of 8 silica beads (2 $\upmu$m diameter). Grabbing a virtual handle the full structure can be rigidly translated, rotated and scaled following hand movements in real time (Fig. 4a). Panel (b) in Fig. 4 shows the phase modulation that is simultaneously displayed on the SLM to generate optical traps at the location dictated by the 3D arrangement of the virtual handle. Panel (c) shows instead the raw holograms recorded on the three color channels of the camera before numerical reconstruction, tracking and rendering on the VR headset. The possibility of using virtual hands to grab objects and to arrange them in 3D configurations that can be inspected immersively and in real time can simplify enormously microassembly tasks, especially for users with no previous experience in microscopy and trapping. In support of this claim, we asked six researchers and students from our University to complete a simple 3D assembly operation consisting of placing a bead mid-point between two other beads. Each user performs the task three times using both our VR interface and a desktop user interface (keyboard or mouse) while the sample is viewed using bright field microscopy on the computer display (See Fig. 4d,e). In both cases we calculate the time to completion as the mean time interval from the moment the trap is created until the trap reaches its target within a tolerance of $0.2\,\upmu$m. The time to completion using VR is significantly shorter than when using the standard interface (p<0.0001) with an overall speed up factor of 6 (Fig. 4f).

**Figure 5:**
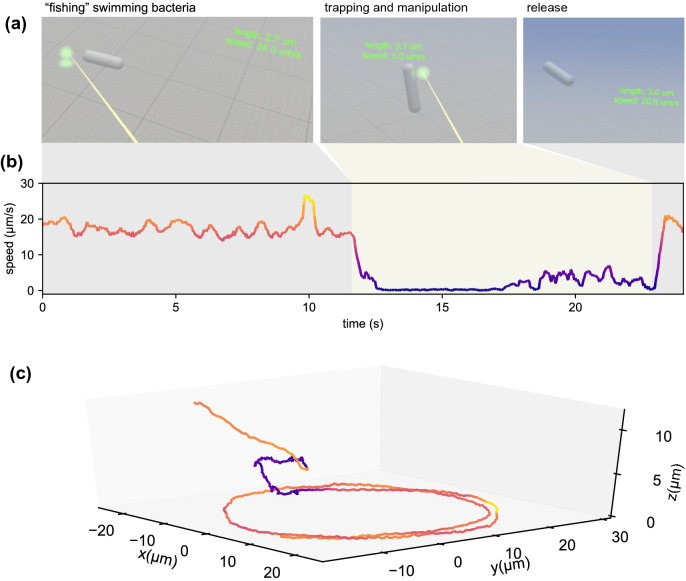
(**a**) The movie frames show the user view as he aims at a swimming *E. coli* cell, traps it and finally releases it. The VR application displays an info panel for each cell showing size and speed. (**b**) Cell speed as a function of time during the interaction summarized by the frames above. (**c**) Full 3D trajectory of the cell showing circular swimming of the free cell, trapping and manipulation (slow speed section) and finally release. The color of the curve corresponds to speed values as shown in (**b**). See also Supplementary Video 3. This figure was made using Unity^28^ and Matplotlib^38^.

### Application example 3: fishing for bacteria

In this last example, we demonstrate that, thanks to the high volume-rate achieved by 3-axis holographic microscopy and the reduced lag times in the information loop running between the holographic and VR engine, we can immersively explore a microenvironment populated by fast swimming bacteria. We implemented an augmented reality system by which looking at a cell triggers the visibility of a panel displaying morphological and dynamical information on the cell such as length and speed (Fig. 5a). This feature could be very useful for future applications when a rapid characterization of motility is important, such as for sperm cells in artificial insemination, that could in addition take advantage of the trapping and sorting capabilities of optical tweezers. The trapping interface for bacteria has been optimized for rapidly moving objects. Colloidal particles diffuse by Brownian motion which gives enough time to navigate in the immediate vicinity of the target bead and grab it with our virtual hands. With a speed of about 20 $\upmu$m/s bacteria can cross the entire field of view (56 $\upmu$m) in less than 3 seconds. We then implemented a virtual laser pointer tool that allows to quickly point at a distant target cell and, when clicking on a trigger, generate an optical trap at the 3D location of the nearest intersection point between the virtual laser pointer beam and the game objects representing real cells. Previously created traps can be also selected with the “pointer” and rapidly moved across the entire field of view “fishing for bacteria” (Fig. 5a). Once a cell falls in a trap we can move it around or bring it closer using the pointer as a virtual “tractor beam” that actually pulls the real optical trap towards the user hand. Last frame in Fig. 5a shows the same cell that, after switching off the traps, is set free and swims away with its initial speed. We can choose to record the tracks of selected objects for subsequent analysis. As an example we show in Fig. 5c the 3D trajectory of the cell during the observation. The plot shows the typical circular trace swept by a swimming cell in presence of a bounding wall^44–46^ (the coverglass in our case). The circles are followed by short trace in which the cell is trapped and a nearly straight line that the cell draws after it is released.

## Discussion

**Figure 6:**
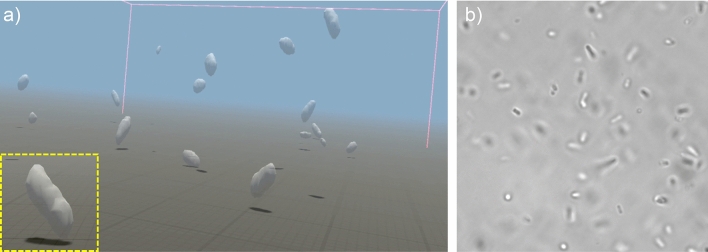
(**a**) Real time mesh rendering of swimming *E. coli* bacteria. The reconstructed volumetric density in the entire field of view is segmented by an isosurface. A close view of an individual cell is shown in the highlighted square panel. (see also Supplementary Video 4). (**b**) For comparison we show a bright field microscopy image of the same bacteria. This figure was made using Unity^28^.

Holographic imaging of objects that, like bacteria, are comparable in size to the wavelength of light is quite a challenging task. At best, volumetric reconstructions represent the convolution of the actual object shape by a point spread function which is approximately a 3D Gaussian and which results in a blurring of the final 3D image (especially along the vertical axis) . For the objects we used in this work, we have a reliable a priori information on the shape: colloids have a spherical shape while bacteria are spherocylinders with a varying length but a pretty constant thickness. In this situation it may be preferable to use volumetric reconstructions to infer the geometric parameters of these shapes and if we render them as perfect spheres and spherocylinders on the VR headset we get a reconstruction of the scene that is closer to reality. However, this is not always the case and there are situations where a more direct and unbiased representation of holographic reconstructions is what we want to see and interact with. To implement that we used a marching cubes algorithm^47^ to obtain isosurfaces of the $512\times 512 \times 61$ reconstructued volumetric images. The routine, executed on the “holographic engine” GPU, outputs a polygonal mesh, whose vertices and triangles are sent over ethernet to the “VR engine” for live rendering. The time taken for marching cubes algorithm to compute all polygons vertices of a given volumetric reconstruction is 3 ms which is faster than the segmentation routine used before. As a benchmark for this imaging modality we demonstrate the real time and immersive exploration of a sample crowded with swimming bacteria (see Fig. 6 and related Supplementary video 4).

A current limitation of our system lies in the difficulty of discriminating nearby objects in conditions of high density. When we propagate scattered fields backwards, interference between scattered fields from nearby objects can give rise to imaging artifacts^23^. For example, our three-axis implementation of DHM fails to return two disconnected regions when two objects are separated by less than 0.5 $\upmu$m in $x-y$ plane. A safer distance along the vertical direction *z* is approximately 1 $\upmu$m.

In conclusion, we have shown that virtual reality provides a powerful interface to merge holographic techniques for 3D microscopy and micromanipulation. The resulting instrument offers an immersive and interactive experience of microscopic phenomena. We can enter our lab on a chip, “walk” on a microscope slide, observe the dynamic phenomena that occur around us in real time and use virtual hands to grab, move and build 3D spatial arrangements of microscopic objects and living cells. This approach can be extended in many different directions. Using tomographic reconstructions we could interact with larger and more complex cells although better algorithms will be required to reach the real time capabilities of the instrument in this paper. Moreover, optical lithography could be brought in as an interactive microfabrication tool where we design our structures using a VR CAD and then monitor the fabrication process in real time by holographic microscopy of the material being photopolymerized.

## Methods

### Computer hardware and software

The VR engine runs on a desktop computer with a TRIX B250F GAMING motherboard, an Intel(R) Core(TM) i7-7700 CPU, 16 gb RAM, a NVIDIA TITAN Xp GPU and Microsoft Windows 10 Pro as the operating system. The holographic engine runs on a desktop computer with a motherboard MPG Z390 GAMING EDGE AC (MS-7B17), an Intel(R) Core(TM) i5-9600K CPU, 32 gb RAM, a GeFrorce RTX 2060 super GPU and a Linux OS (Ubuntu 20.04.1 LTS). We used Unity^28^ as the game engine and the VR system Oculus Rift CV1. Game objects in the Unity application implement clients that connect to two separate UDP sockets running on the holographic server and managing data transfer for trap coordinates and sample geometric parameters.

### Optical hardware

RGB light sources: Prizmatix UHP-T-LED-460, UHP-T-LED-520 and UHP-T-LED-630. Spatial Light Modulator: Hamamatsu X10468-03. Camera: Basler avA1000-100gc.

### Sample preparation

Bacteria of the strain RP437^48^ (wild type) and HCB437^49^ (smooth swimmer) were grown overnight in 10 mL of LB supplemented with 100 $\upmu$M ml$^{-1}$ streptomicyn or 30 $\upmu$M ml$^{-1}$ kanamicyn respectively, at 30 $^\circ$C, 200 rpm. In the morning the culture was diluted 1:100 in 5 mL of Tryptone Broth (1% Tryptone, 0.5% NaCl) with kanamicyn or streptomicyn and grown at 30 $^\circ$C, 200 rpm until an OD600 of 0.6-0.8. Then the cells were washed through centrifugation three times at 1500 g in motility buffer (67 mM NaCl, 10 mM potassium phosphate, 0.1 mM EDTA, 10mM glucose, 0.02% Tween20 at pH 7.0).

## Supplementary Information

Supplementary Information 1.

Supplementary Information 2.

Supplementary Information 3.

SupplSupplementary Information 4.

Supplementary Information 5.
