# Supplementary material for: A virtual reality interface for the immersive manipulation of live microscopic systems
Source: Sci Rep. 2021 Apr 7;11:7610. doi: 10.1038/s41598-021-87004-5 (PMC8027422; doi:10.1038/s41598-021-87004-5)
Supplement: Supplementary file 1 — Supplementary Information 1. [file 41598_2021_87004_MOESM1_ESM.docx]

**Supplementary file for**

**Virtual micro-reality: immersive manipulation of live microscopic systems**

**Silvio Bianchi**^1^**, Stefano Ferretti**^2^**, Giacomo Frangipane**^2,1^**, and Roberto Di Leonardo**^2,1^

^1^ NANOTEC-CNR, Institute of Nanotechnology, Soft and Living Matter Laboratory, Roma I-00185, Italy

^2^ Sapienza University of Rome, Physics Department, Roma I-00185, Italy

**Supplementary Video 1**

An immersive falling body experiment at the micron scale. The user grabs and lifts two microspheres made of glass (right) and polystyrene (left). After release the spheres are observed while sedimenting with different average speed as evidenced by the info panel displaying particles heights.

**Supplementary Video 2**

Live and immersive micromanipulation of colloidal beads showing assembly of 3D microstructures and subsequent rigid manipulation through a virtual handle.

**Supplementary Video 3**

Fishing for bacteria using a virtual laser pointer to target a swimming cell and capture it in an optical trap. The cell swims away when the trap is switched off.

**Supplementary Video 4**

Live immersive exploration of a dense sample of swimming bacteria directly rendered via an isosurface mesh of volumetric reconstructions.
